# Vitamin A – a scoping review for Nordic nutrition Recommendations 2023

**DOI:** 10.29219/fnr.v67.10229

**Published:** 2023-11-07

**Authors:** Thomas Olsen, Ulf H. Lerner

**Affiliations:** 1 Department of Nutrition, Institute of Basic Medical Sciences, University of Oslo, Oslo, Norway; 2 Department of Internal Medicine and Clinical Nutrition, Institute for Medicine, Sahlgrenska Academy at University of Gothenburg, Gothenburg, Sweden

**Keywords:** vitamin A, retinol, carotenoids, retinol equivalents, dietary recommendations

## Abstract

Vitamin A refers to a group of fat-soluble compounds with retinol activity, including all-*trans* retinol and pro-vitamin A carotenoids. Bioactive compounds include retinal and all-*trans* retinoic acid with important functions in vision, immune function, growth, and development. The literature search that was performed for the current scoping review yielded a total of seven publications relevant to setting the recommended daily intake for vitamin A. In total, six publications assessed the relationship of serum retinol and/or dietary vitamin A intake with fracture risk (*n* = 2), cancer (*n* = 3), and deficiency after bariatric surgery (*n* = 1). One additional report by the European Food Safety Administration (EFSA) with updated average requirements was included. The outcomes-based systematic reviews and meta-analyses showed positive associations for vitamin A intake and serum retinol with risk of hip fracture. Weak or inconclusive associations were observed for cancer or obesity. One publication by EFSA with updated estimated average requirements and population reference intakes for dietary vitamin A intakes was published in 2015. The EFSA recommendations and estimated average requirements are based on a European reference population, with body weights derived from an assumed body mass index of 22, which might be too low and not representative of the Nordic and Baltic populations, and consequently resulting in lower estimated average requirements and recommendations. In conclusion, there were limited new outcomes-based data for vitamin A and health outcomes.

## Popular scientific summary

- Vitamin A refers to a group of fat-soluble compounds with retinol activity, including all-*trans* retinol and pro-vitamin A carotenoids.
- Retinol is considered the principle form of the vitamin A, while retinal and retinoic acid are the active forms of vitamin A involved in the visual process in the retina and transcription regulation in almost all cell types, respectively.
- Dietary intake needed to maintain a liver retinol concentration of 20 μg/g liver is used as indicator to set vitamin A requirement.
- Deficiency is defined as liver retinol concentrations <0.07 μmol/g or serum/plasma retinol <0.7 μmol/L.
- Clinical deficiency manifests as xerophtalmia and impaired resistance to infection.
- Recommended intake of vitamin A is expressed in terms of retinol equivalents (REs).

Vitamin A refers to a group of essential fat-soluble compounds with retinol activity including pro-vitamin A carotenoids (1). Vitamin A can be obtained from both animal and plant sources in the diet (1). In animal sources, vitamin A exists predominantly as retinyl palmitate (a retinyl ester), whereas in plants, it exists only in the form of pro-vitamin A carotenoids such as β-carotene. β-Carotene is one of 50–60 members of a large class of naturally occurring compounds called carotenoids that have retinol activity, implicating that at least one intact molecule of retinol or retinoic acid can be obtained from the carotenoid.

The major circulating form is all-*trans*-retinol, which can be converted to the hormonally active compound, all-*trans-*retinoic acid (ATRA), in the liver and other target tissues, where it exerts its biological activity through nuclear receptors (1). Vitamin A is also crucial for nighttime vision as part of the photopigment rhodopsin in the eye. Here, 11-*cis* retinal is the major bioactive component that is crucial for rhodopsin formation. Vitamin A can be stored in the body and can therefore protect from vitamin A insufficiencies for several months. The main storage form of vitamin A is as retinyl esters formed by esterification of retinol in the liver.

Recommendations on vitamin A are formulated based on sources of preformed vitamin A (retinol and retinyl esters) and conversion of pro-vitamin A carotenoids, including α-carotene, β-carotene, and β-cryptoxanthin. To convert all sources of vitamin A into a single unit, the terms ‘retinol equivalents’ (RE) or ‘retinol activity equivalents’ (RAE) are used. The term RE is used by the WHO/FAO and EFSA, whereas the term RAE is used by the US National Academy of Medicine (previously Institute of Medicine (IOM)), and the differences lie in estimated conversion factors of β-carotene and other carotenoids to retinol. The WHO/FAO and EFSA estimate that 6/12 μg of dietary β-carotene/other carotenoids are required to produce 1 μg of retinol (2, 3), whereas the IOM estimates that 12/24 μg of β-carotene/other carotenoids are required to produce 1 μg of retinol (4). There is still uncertainty about the appropriate bioconversion factor applied for β-carotene to retinol. The absorption and bioconversion of β-carotene vary considerably, and Haskell reported that 5–65% of β-carotene is bioavailable depending on the source (5). Haskell further reported equivalencies ranging from 2 to 55:1 in oil with a median of 3.7:1 and ranging from 4.5 to 27:1 in plant foods with a median of 12.5:1. In another review, Von Loo-Bouwman et al. reported equivalencies in oils ranging from 2 to 55:1 with a median of 3.5:1, whereas a range of 5.4–28:1 was reported for plant foods with a median of 12.5:1 that depended on population and source and food matrix (6). In a recent compartmental modeling study that aimed to describe whole-body β-carotene metabolism, Green et al. used data from healthy older adults that ingested 6 mg labeled β-carotene and reported vitamin A equivalencies ranging from 6.6 to 15:1 μg β-carotene:μg retinol (7), with a median of 13:1.

Present evidence does not allow us to accurately estimate the correct conversion factor for β-carotene. As discussed, there are arguments that both favor a conversion factor of 6:1 and 12:1. Until more evidence is published, and in order to be consistent with recent legislation in most European countries, we use a conversion factor of 6:1 for all sources of β-carotene in the present NNR, and the term RE will be maintained. Thus, the conversion factors will be as follows:

- 1 μg of dietary or supplemental preformed vitamin A (i.e. retinol)
- 2 μg of supplemental β-carotene
- 6 μg of dietary β-carotene
- 12 μg of other dietary pro-vitamin A carotenoids (e.g. α-carotene and β-cryptoxanthin)

## Methods

The scoping review follows the protocol developed within the NNR2023 project (Box 1) (8). The sources of evidence used follow the eligibility criteria described in the paper ‘The Nordic Nutrition Recommendations 2022 – Principles and methodologies’ (9). One qualified systematic review (qSR) published after 2012 was identified in the initial scoping review on vitamin A by the NNR 2023 committee (2). For the present scoping review authors’ literature search, the search string used was (((((‘vitamin a’[Title])) AND (review[Publication Type] OR systematic review[Publication Type] OR meta-analysis[Publication Type])) AND (‘2011’[Date – Publication] : ‘3000’[Date – Publication]))) AND Humans[Filter]. The search yielded 226 hits. Based on title and abstract screening, 11 reviews were considered relevant for either setting new dietary reference values (DRVs) for vitamin A or relevant to health outcomes in the Nordic or Baltic countries. None fulfilled the set of criteria set down by the NNR committee for qSRs (10). However, six systematic reviews were considered relevant for the sub-chapters cancer (11–13), osteoporosis and fractures (14, 15), and obesity (16), and their results are briefly discussed in this scoping review. These six reviews were quality checked with the AMSTAR2-NNR tool and were considered of low or critically low quality (Table 1). Also, the scarcity of data did not always allow appropriate methods for meta-analysis. Despite this, these reviews address highly relevant research questions concerning dietary and circulating vitamin A and will be discussed briefly. An updated search was performed on August 1st, 2022. The scoping review authors and the NNR committee are aware of these publications; however, the information in these publications does not change the judgment regarding the setting of DRVs/Food-based dietary guidelines.

Box 1 General information

- This paper is one of many scoping reviews commissioned as part of the Nordic Nutrition Recommendations 2023 (NNR2023) project (Blomhoff et al, NNR report (8))
- The papers are included in the extended NNR2023 report, but, for transparency, these scoping reviews are also published in Food & Nutrition Research.
- The scoping reviews have been peer reviewed by independent experts in the research field according to the standard procedures of the journal.
- The scoping reviews have also been subjected to public consultations (see report to be published by the NNR2023 project).
- The NNR2023 committee has served as the editorial board.
- While these papers are a main fundament, the NNR2023 committee has the sole responsibility for setting dietary reference values in the NNR2023 project.

**Table 1:** Appraisal of systematic reviews identified in the literature search

*Domain* ^*^	Zhang (13)	Wu (14)	Zhang (15)	He (11)	Leelakanok (12)	Lewis (16)
1	Yes	Yes	Yes	Yes	Yes	Yes
2^**^	No	No	No	No	No	No
3^**^	Yes	Yes	Yes	Yes	Yes	Yes
4	No	Yes	Yes	Yes	No	Yes
5	Yes	Yes	Yes	Yes	Yes	Yes
6	No	No	No	No	No	No
7	Yes	Yes	No	Yes	Yes	Yes
8^**^	Yes	Yes	Yes	Yes	Yes	Yes
9	No	No	No	No	No	No
10^**^	Yes	Yes	Yes	Yes	Yes	N/A
11^**^	Yes	No	No	No	Yes	Yes
12	Yes	No	Yes	Yes	Yes	Yes
13^**^	Yes	Yes	Yes	No	Yes	N/A
14	Yes	Yes	Yes	Yes	Yes	Yes

Overall confidence	Critically low	Critically low	Critically low	Critically low	Critically low	Low

* AMSTAR2-NNR Domains: 1: Did the research questions and inclusion criteria for the review include the components of PICO? 2: Did the report of the review contain an explicit statement that the review methods were established prior to the conduct of the review and did the report justify any significant deviations from the protocol? 3: Did the review authors use a comprehensive literature search strategy? 4: Did the review authors perform study selection in duplicate? 5: Did the review authors perform data extraction in duplicate? 6: Did the review authors provide a list of excluded studies and justify the exclusions? 7: Did the review authors describe the included studies in adequate detail? 8: Did the review authors use a satisfactory technique for assessing the risk of bias (RoB) in individual studies that were included in the review? 9: Did the review authors report on the sources of funding for the studies included in the review? 10: If meta-analysis was performed, did the review authors use appropriate methods for statistical combination of results? 11: Did the review authors account for RoB in individual studies by subgroup analysis, or when they interpreted/discussed the results of the review? 12: Did the review authors provide a satisfactory explanation for, and discussion of, any heterogeneity observed in the results of the review? 13: If they performed quantitative synthesis, did the review authors carry out an adequate investigation of publication bias (small study bias) and discuss its likely impact on the results of the review? 14: Did the review authors report any potential sources of conflict of interest, including any funding they received for conducting the review?

** AMSTAR2-NNR critical domains.

## Physiology

**Fig. 1:**
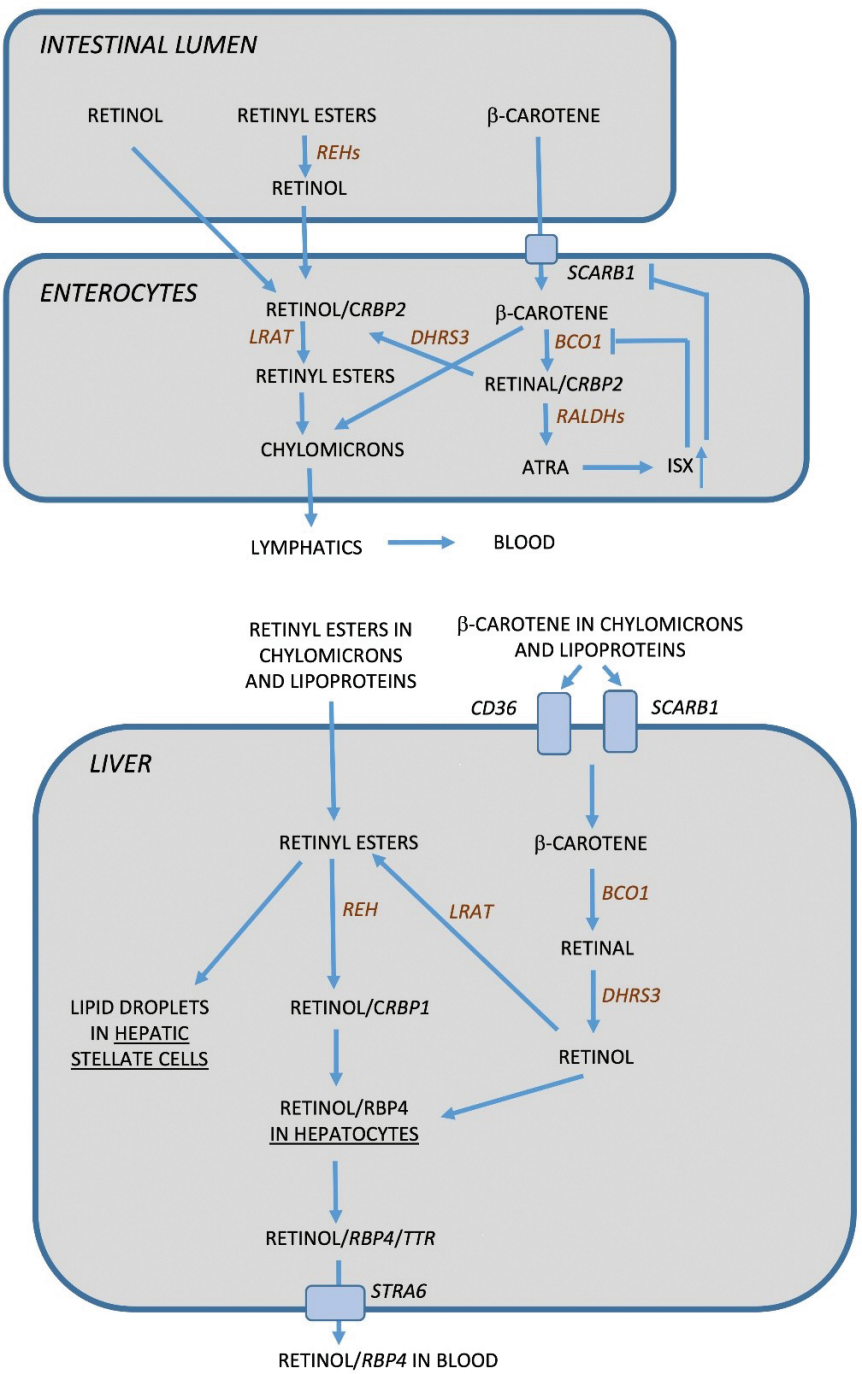
In the intestinal lumen, retinyl esters are converted to retinol by REH (retinyl ester hydrolase). Retinol and β-carotene in the intestine is taken up by enterocytes where retinol bound to CRBP2 (cellular retinol-binding protein 2) is converted to retinyl esters through LRAT (lecithin:retinol acyltransferase) and packed in chylomicrons. β-carotene in enterocytes is either packed in chylomicrons or converted to retinal through BCO1 (β-carotene 15,15´-dioxygenase); retinal bound to CRBP2 is then converted to retinol through DHRS3 (NADPH-dependent dehydrogenase reductase 3) and subsequently to retinyl esters through LRAT and stored in chylomicrons. As a feedback mechanism, ATRA is formed from retinal through a two-step process by RALDHs (retinal dehydrogenases) to induce the expression of ISX (intestinal-specific homeobox gene) which inhibits uptake of β-carotene through SCARB1 (scavenger receptor class B type 1) and the conversion to retinal through BCO1. Chylomicrons are released into lymphatics and subsequently to the blood. Circulating retinyl esters present in chylomicrons and bound to lipoproteins are taken up by liver cells where it is either stored in stellate cells or converted to retinol and bound to RBP4 (retinol binding protein 4) and TTR (transthyretin). In the liver cells, β-carotene in chylomicrons and bound to lipoproteins are taken up by CD36 and SCARB1 and subsequently converted to retinal by BCO1 and then to retinol through DHRS3. Retinol is then either converted to retinyl esters to be stored in stellate cells or bound to RBP4 and TTR to be released to the blood. Retinol released from the liver is present in the circulation as retinol bound to RBP4.

Prior to uptake, retinyl esters from animal sources are converted to retinol by REH (retinyl ester hydrolase) in the intestinal lumen. The absorption of ingested retinol and ß-carotene in the intestine is facilitated by co-ingestion of fat (17, 18). The dietary fat stimulates secretion of bile salts, which causes emulsification of retinol in micelles, thereby increasing the absorption of retinol (19). Retinol and carotenoids are taken up from the lumen by enterocytes, in which retinol bound to CRBP2 (cellular retinol-binding protein 2) is esterified to retinyl esters by LRAT (lecithin:retinol acyltransferase) (20) (Fig. 1). β-Carotene from plant-based foods is transformed in enterocytes to retinal by BCO1 (β-carotene 15,15´-dioxygenase). Retinal bound to CRBP2 is subsequently converted to retinol by DHRS3 (NADPH-dependent dehydrogenase reductase 3) and finally to retinyl esters by LRAT. The retinyl esters, together with some amounts of β-carotene, are packed into chylomicrons, which are transported to the lymphatics and subsequently released into the blood, of which 70% are delivered to the liver and the rest to target cells. In the circulation, both retinyl esters and β-carotene are also present in association with lipoproteins such as very low-density lipoproteins. Small amounts of retinal in enterocytes are metabolized to ATRA by RALDHs (retinal dehydrogenases) (see further below). Interestingly, ATRA induces the expression of the transcription factor ISX (intestinal-specific homeobox gene), which represses the expression of BCO1. ISX also inhibits the expression of SCARB1 (scavenger receptor class B type 1), an intestinal transporter protein important for the uptake of β-carotene into enterocytes. The ATRA-induced control of the expression of both these genes acts as feedback mechanism to suppress the conversion of β-carotene to retinol, which explains why toxicity to β-carotene does not occur (21, 22).

Retinyl esters present in the chylomicrons, chylomicron remnants or lipoproteins are delivered to hepatocytes in the liver, and the retinyl esters are then either stored in lipid droplets in hepatic stellate cells or hydrolyzed to retinol by REH and then bound to CRBP1 (23–25) (Fig. 1). This complex is transferred to hepatocytes, where retinol is bound to RBP4 (retinol-binding protein 4) and to the transporter protein TTR (transthyretin) and subsequently released to the blood. The cellular uptake of β-carotene in the liver is facilitated by the scavenger receptors SCARB1 and CD36 (also known as SCARB3 (Fig. 1)). In the cytosol, β-carotene is then converted to retinal by BCO1 and then reduced to retinol by DHRS3. Retinol thus formed can then either be esterified by LRAT to retinyl esters or bound to RBP4 and secreted to be used by target cells.

In the circulation, retinoids mainly exist not only as retinol bound to RBP4 but also as retinyl esters and β-carotene in chylomicrons and other lipoproteins (Very low-density lipoprotein, low-density lipoprotein, and high-density lipoprotein) if concentrations are very high, or as ATRA bound to albumin. In the fasting state, the concentration of retinyl esters in plasma is 100–200 nM but increases to 5–10 μM after a retinoid rich meal; after which, it serves as the main source for cellular uptake (25). In the fasting state, the concentrations of retinol/RBP4 and β-carotene are 2–4 and 5–8 μM, respectively. During this state, retinol/RBP4 replaces retinyl esters as the main source for cellular uptake. The plasma concentration of ATRA is generally low and varies from 1 to 3 nM during fasting state and increases to 80–90 nM in the postprandial phase.

**Fig. 2:**
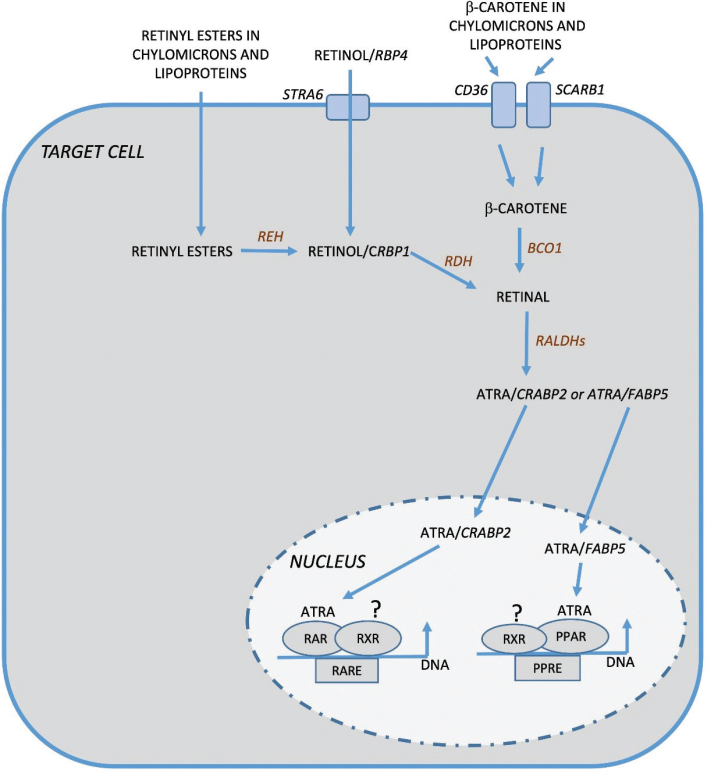
In target cells, retinol is taken up through STRA6 (stimulated by retinoic acid 6) and bound to CRBP1 (cellular retinol-binding protein 1) and subsequently oxidized to retinal by RDH (retinol dehydrogenase). Retinyl esters present in chylomicrons and lipoproteins are taken up and also serve as cellular source for conversion to retinol and retinal. In addition, β-carotene in chylomicrons and lipoproteins is taken up through CD36 and SCARB1 (scavenger receptor class B type 1) and then converted to retinal by BCO1 (β-carotene 15,15´dioxygenase). Retinal is finally metabolized by two different RALDHs (retinal dehydrogenases) to ATRA. ATRA bound to CRABP2 (cellular retinoic acid binding protein 2) is shuttled to the nucleus and then ligated to retinoic acid receptors (RARs). ATRA can also be bound to FABP5 (fatty acid-binding protein 5) and is then ligated to PPAR (peroxisome proliferator-activated receptorβ/δ). Both RARs and PPARβ/δ heterodimerize with retinoic X receptors (RXRs). These complexes cause activation or repression of gene transcription through RARE (retinoic acid response elements) or PPRE (PPAR response elements), respectively.

In target cells, retinol/RBP4 is taken up by STRA6 (stimulated by retinoic acid 6) and β-carotene present in chylomicrons and other lipoproteins by the scavenger proteins CD36 and SCARB1 (24, 26) (Fig. 2). Retinyl esters in chylomicrons and other lipoproteins, and ATRA bound to albumin, also serve as sources for uptake of retinoids by target cells. Retinol delivered through STRA6, or formed by the conversion of retinyl esters by REH, is bound to CRBP1 and then oxidized by retinol dehydrogenase (RDH) to retinal. Retinal is also formed from β-carotene by BCO1. Retinal is converted by a two-step oxidation process through RALDHs to ATRA. ATRA bound to CRABP2 (cellular retinoid acid-binding protein 2) is shuttled to the nuclei, where it binds to retinoid nuclear receptors (RARs). Similar to other nuclear receptors, RARs in complex with ATRA heterodimerize with retinoic X receptors (RXRs) and function as ligand-dependent transcription factors. FABP5 (fatty acid-binding protein 5) can also bind ATRA facilitating the binding of ATRA to PPARβ/δ (peroxisome proliferator-activated receptor β/δ), which is another form of nuclear receptors able to form heterodimers with RXRs (27).

Two families of nuclear receptors mediate the classical biological effects of retinoids, retinoic acid receptors (RARs), and retinoic X receptors (RXRs) (25, 28, 29). Three isotypes (α, β, and γ), encoded by separate genes, make up each family, and these receptor subtypes are present in different isoforms due to alternate splicing and different promoter usage. The fact that gene sequence for each isotype differs significantly, but is highly conserved between humans and mice, has given rise to the speculation that each RAR isotype has specific functions. When ATRA binds to RAR, the receptors heterodimerize with RXR and subsequently undergo conformational changes in the ligand-binding domain of the receptor, making it possible to replace corepressors with coactivators and to recruit histone acetyltransferases and methyltransferases (Fig. 2). The activated RAR/RXR complex can then bind to RAREs (retinoic acid response elements) in gene regulatory regions upstream of a variety of genes, which will either cause activation or repression of gene transcription. The ligand for RXRs has been elusive for many years but it seems that 9-cis-13,14-dihydroretinoic acid may be a physiologically relevant ligand for RXR. ATRA can also act as an alternative ligand for PPARβ/δ and be directed to the RXR/PPARβ/δ dimer, which is then recruited to PPREs (PPAR response elements) in different gene regulatory regions and thereby function as another mechanism by which ATRA can regulate transcription (30). RXRs can also induce transactivation or transrepression by interacting with other nuclear receptors or transcription factors (25, 31).

For the visual functions of vitamin A in the eye, retinyl esters are metabolized to 11-cis-retinal through the enzymatic activity of the retinoid isomerohydrolase RPE65. Together with opsin, 11-cis-retinal makes up the chromophore rhodopsin. Using light energy and photoisomerization, rhodopsin is decomposed to opsin and all-trans-retinal, resulting in a visual signal from retina to the central nervous system.

Vitamin A has numerous important physiological functions in the body not only during embryonic development but also during postnatal life, including making up the light sensitive chromophore rhodopsin in eyes. Numerous genes contain RAREs, and the transcriptional activity includes regulation of genes involved in immunity, reproduction, maintenance of epithelial functions, maintenance of epithelial surfaces, immune competence, growth, and bone remodeling (25).

## Assessment of vitamin A status

Depending on the population, assessment of vitamin A status can be done using dietary assessment methods and physiological and biochemical indicators. Physiological indicators include assessment of dark adaptation in suspected deficiency, whereas biochemical indicators include serum RBP4 and retinol in serum, dried blood spot and breast-milk retinol, retinyl esters in serum, relative dose-response tests, and isotope dilution techniques (32). The traditional cut-off for vitamin A deficiency is <0.07 μmol retinol/g liver (20 μg retinol/g liver) (2), which is the minimum liver reserve used for the estimation of vitamin A requirements. The latest Biomarkers of Nutrition and Development (BOND) publications call for this cut-off to be increased to <0.1 μmol retinol/g liver (32, 33). Because liver tissue is unavailable in humans, serum retinol and/or RBP4 is commonly used to assess vitamin A status. The generally accepted definition of deficiency measured as serum/plasma retinol at the population level is <0.7 μmol/L, whereas others have proposed to use <1.05 μmol/L (2, 32, 34). It has been argued that a cut-off at 1.05 may lead to overestimation of vitamin A deficiency (34), and, thus, we proceed with <0.7 μmol/L as the definition of deficiency. For liver stores, adequacy is defined as ranging from 0.1 to 0.7 μmol retinol/g liver (32).

Although serum retinol and RBP4 are considered adequate to assess vitamin A deficiency on a population level, interpretation of serum concentrations in affluent societies can be challenging for a variety of reasons. For example, retinol is kept under tight homeostatic control and generally does not respond to dietary intakes unless they remain deficient or extremely excessive over time (35). Both conditions are relatively rare in Western populations. In addition, retinol is sensitive to inflammation, which can lead to underestimated levels (36). For RBP4, this marker is thought to be increased in overweight and obesity (37) and is an adipokine with properties independent of retinol transport protein (38). Using RBP4 as a measure of status can thus lead to overestimation of vitamin A status in individuals with overweight and obesity. Collectively, although serum retinol and RBP4 are useful markers in the context of deficiency, they lack sensitivity for assessment in areas where vitamin A status is generally considered sufficient.

As liver biopsies are generally not available from humans, an adequate measure of vitamin A status is using isotope dilution techniques, which is thought to reflect total body vitamin A stores in the absence of inflammation (32). However, this procedure is rarely carried out when assessing retinol status in developed countries, and we are not aware of any such data from Nordic or Baltic populations. One recent study in US women showed that mean ± standard deviation liver vitamin A concentrations, as measured by isotope dilution techniques, were 0.45 ± 0.31 μmol/g liver (39). When supplement users were excluded, hepatic and total body vitamin A stores did not correlate with self-reported vitamin A intakes, and thus, assessment of status remains a challenge in nutritionally vitamin A replete populations. Notably, intakes around 500 RE/d predicted liver stores four times the minimal cut-off on which the estimated average requirements in the US guidelines are based.

## Vitamin A intake in the Nordic and Baltic countries

Dietary intakes of vitamin A have been summarized in the background review on dietary intake in the Nordic and Baltic countries and published in the background paper by Lemming & Pitsi (40). Briefly, vitamin A intakes were higher than the recommended intakes from NNR 2012 in both the Nordic and Baltic countries. Average intake of vitamin A ranged from 812 RE/d among men in Sweden to 1,556 RE/d among men in Denmark, and from 747 RE/d among women in Finland to 1,110 RE/d among women in Denmark. In the Baltic countries, average intake ranged from 1,053 RE/d among men in Lithuania to 1,155 RE/d among men in Estonia. For women, average intake ranged from 934 RE/d in Lithuania to 942 RE/d in Estonia. In Latvia, average intake was reported jointly for males and females across ages 7–64 and was 666 RE/d.

Data from children and adolescents in various age groups were available from Denmark (41), Finland (42), Iceland (43), Norway (44), Sweden (45), Estonia (46), and Lithuania (47). In age groups 2–5, 6–9, and 10–13 years, recommendations from NNR2012 were 350, 400, and 600 RE/d, respectively. Notably, in Denmark, average vitamin A intakes were 1,271 (boys) and 1,096 (girls) RE/d among 4–5 year olds; 1,236 (boys) and 1,210 (girls) RE/d among 6–9 year olds; and 1,192 (boys) and 943 (girls) RE/d among 10–13 year olds. In other words, vitamin A intake among Danish 2–9 year olds was well in excess of recommended intakes. The main source of vitamin A in the Danish population as a whole is meat (40%) and vegetables (24%) (41). Children in the other Nordic and Baltic countries were also in excess of recommended intakes, but not to the same extent as in Denmark, with the exception of Estonian 6–9 year old boys with intakes of 1,136 RE/d.

## Health outcomes relevant for Nordic and Baltic countries

### Deficiency

There is some variability in the definitions of deficiency. Vitamin A deficiency is defined as liver stores of <0.07 or <0.10 μmol retinol/g liver depending on the publication, or alternatively serum/plasma retinol of <0.7 μmol/L (2, 32–34). When the intake of vitamin A is inadequate to meet physiological needs, clinical vitamin A deficiency develops and is characterized by several ocular features (xerophthalmia) and impaired resistance to infection, and increased infectious disease mortality. According to the WHO, ocular features have been reported for plasma/serum retinol concentrations of 0.35 μmol/L (48). Low liver stores as assessed by modified relative dose-response tests were observed in Indonesian women with a serum retinol of <0.7 μmol/L (49). Vitamin A deficiency is probably uncommon in developed countries compared to developing countries (32, 50) but might be under-diagnosed because there is a lack of simple screening tests to measure subclinical deficiency as circulating retinol is kept under tight homeostatic control (35).

### Toxicity

Chronic or acute hypervitaminosis A (>1 μmol retinol/g liver) leads to clinical symptoms, including nausea, vomiting, skin disorders, and liver damage (2, 32). Excessive intakes in pregnancy are teratogenic. In NNR2012, tolerable upper intake level was set to 3,000 RE/d of preformed retinol for adults (not adapted to children). EFSA set tolerable upper intake levels for children and adolescents ranging from 800 to 1,600 RE/d depending on their age. Of note, Danish children in the youngest age group exceed the tolerable upper intake levels reported in EFSAs recommendations (see review on dietary intakes in NNR). It has been suggested that intake even marginally above the recommended dietary intake is associated birth defects, reduced bone mineral density, and increased risk for hip fracture (see below).

### Fracture risk and osteoporosis

Numerous studies in mice and rats have demonstrated that high doses of vitamin A cause harmful effects mainly on cortical bone due to excessive formation of periosteal osteoclasts, resulting in diminished cortical width and in some cases spontaneous fractures (51, 52). Most of these studies, however, have been performed with high, intoxicating doses of vitamin A (13 to at least 142 times higher than the recommended dietary allowance (RDA)) given during short time periods (7–14 days) (53). Studies using lower (subclinical) doses of vitamin A (50 times higher than controls) for 12 weeks resulting in a 20-fold increase of serum retinyl esters (5.25 μmol/L) demonstrated decreased cortical thickness and width, resulting in enhanced bone fragility (54). A recent study showed that even lower doses of vitamin A, close to the upper tolerable levels in humans (4.4x higher than controls) and resulting in serum levels of retinyl esters of 0.14 μmol/L, resulted in decreased cortical bone mass when given to mice during longer time periods (10 weeks) (53).

Pioneering studies in Sweden, based upon a cross-sectional study in 175 women aged 28–74 years, showed that dietary intake of retinol >1.5 mg/d was positively associated with significantly decreased bone mineral density at several skeletal sites, including femoral neck, lumbar spine, and proximal femur, when compared to a dietary intake of <0.5 mg/d (55). Using a nested control–study with 247 women experiencing a hip fracture, the authors also reported that the risk for hip fracture was doubled in individuals with a dietary intake of retinol >1.5 mg/d, when compared to 873 age-matched controls. Dietary intake of β-carotene was not associated with bone mineral density or fracture risk. Subsequent studies confirmed the positive association between high dietary intake of retinol and decreased bone mineral density and increased risk for hip fracture (56, 57). In a population-based, longitudinal cohort study with 2,047 men and a follow-up time of 30 years, Michaelsson et al. for the first time reported that the overall risk of fracture (*n* = 266) was positively related also to serum levels of retinol, with largest risk concentrated in the highest quantile for serum retinol (>2.64 μmol/L) (58).

Since then, many studies have been performed to investigate whether these studies can be confirmed in other cohorts, but the results are divergent. Some studies have reproduced the initial results reporting a detrimental effect by vitamin A on bone, but others have found either no association or a beneficial effect (51, 52, 59).

The literature search conducted for this scoping review yielded two systematic reviews and meta-analyses for vitamin A and risk of fracture and osteoporosis. One meta-analysis assessed the effect of vitamin A on risk of hip or total fractures (14). The analyses included eight prospective studies, in which the intake of total vitamin A, retinol, or β-carotene was available, and four studies, in which serum retinol had been analyzed. High intake of total vitamin A or retinol, but not β-carotene, increased the relative risk of hip fracture but not that of total fracture. Both high and low levels of serum retinol were associated with increased risk of hip fracture without affecting the risk of total fracture. In another meta-analysis, high intake of retinol or total vitamin A slightly increased the risk of hip fracture (15). However, increased serum levels of retinol were not significantly associated with hip fracture risk. In agreement with the other meta-analysis, low serum levels of retinol slightly increased hip fracture and total fracture risk.

### Cancer

One systematic review and meta-analysis including 10 studies showed that vitamin A intakes were not associated with all-cause mortality among pre-diagnostic or post-diagnostic breast cancer patients (11). Another systematic review and meta-analysis of five studies found an inconclusive association between serum retinol and liver cancer (12). In a systematic review and meta-analysis of 11 studies on the risk association between vitamin A intake and cervical cancer, dietary total vitamin A intake and its components (retinol, β-carotene, and other carotenoids) were all inversely associated with cervical cancer (13). For blood retinol, a positive but inconclusive association was found with risk of cervical cancer, whereas blood β-carotene was significantly and inversely associated with cervical cancer risk.

### Obesity and diabetes

Elevated concentrations of the transport protein of retinol in plasma, RBP4, have been identified as an adipokine with specific roles in adiposity and impaired insulin sensitivity. The literature search described in the methods section yielded several reviews on molecular mechanisms, by which retinoids and RBP4 may influence energy metabolism, insulin sensitivity, and adiposity. However, this concept remains poorly understood, and there are currently no systematic reviews in humans linking these findings to vitamin A intake or serum retinol status. However, one systematic review evaluated the effect of bariatric surgery on micronutrients status (16); evidence was inconsistent with regard to vitamin A deficiency among these patients.

## Requirement and recommended intake

Vitamin A recommendations are based on the required intake to maintain liver retinol concentrations of 20 μg retinol/g liver. NNR2012 was based on IOM 2001 (4) and yielded estimated intakes of 700 RE/d for women and 900 RE/d for men. After applying body weight extrapolations, recommendations were set to 350 RE/d for children of 2–5 years, 400 RE/d for children of 6–9 years, and 600 for children of 10–13 years. To account for increased demands in pregnancy and lactation, recommendations were set to 800 and 1,100 RE/d, respectively.

The initial literature search performed by the NNR 2023 committee identified updated guidelines for vitamin A published in 2015 (2). The population reference intakes in EFSA were set to 750 RE/d for men and 650 RE/d for women. EFSA recommends slightly higher intakes of vitamin A for pregnant (700 RE/d) and lactating women (1,300 RE/d) compared to non-pregnant women based on the accumulation of vitamin A in the fetus and loss of retinol in breast milk (2).

EFSA recommends the following dietary vitamin A intakes for specific age groups after body weight adjustments:

- 7–11 months: 250 RE/d
- 1–3 years: 250 RE/d
- 4–6 years: 300 RE/d
- 7–10 years: 400 RE/d
- 11–14 years: 600 RE/d
- 15–17 years (males): 750 RE/d
- 15–17 years (females): 650 RE/d

### Approaches to estimating average requirements

Both IOM 2001 and EFSA 2015 use the factorial approach to estimate vitamin A requirements, as listed in Table 2. Factors A through G are multiplied to arrive at average requirements that are, in turn, multiplied by coefficients of variation to yield final recommendations. There are some crucial differences in the two approaches that warrant discussion: NNR2012 was based on the IOM 2001 approach, which was based on a North American reference population. EFSA used more recent data on body/liver stores of vitamin A catabolic rates but relies on Olson (37) for the calculation of fractional catabolic rate. The two approaches also use differing CVs to account for variation. The results from multiplying the different equation components yield clear differences in estimated average requirements and consequently recommendations. These differences are mainly explained due to the different reference body weights used in the respective approaches. Notably, using the IOM reference body weight in the EFSA approach would yield similar average requirements and recommended intakes. A notable distinction here is that EFSA uses an approach that assumes a body mass index (BMI) of 22 kg/m^2^, resulting in substantially lower body weights that poorly reflect the actual BMI in their reference population. Of note, the median measured body weights range from 75 to 82 kg and from 60 to 68 kg for men and women, respectively, across age groups, closer to the IOM 2001 reference body weights. Thus, basing vitamin A recommendations on a body weight derived from an ‘optimal’ BMI of 22 is not necessarily representative for the Nordic and Baltic populations. For instance, about 25% of men and 20% of women in middle age are overweight or obese in Norway (60), which could be considered when giving recommendations.

**Table 2:** Factorial approaches used by IOM 2001 and EFSA 2015 to estimate average requirements and recommended intakes of vitamin A

	IOM 2001	EFSA 2015
A) Target liver concentration (μg retinol/g)	20	20
B) Body/liver retinol stores	1.1	1.25
C) Liver/body weight ratio	0.030	0.024
D) Fractional catabolic rate (%)	0.005	0.007
E) 1/Efficiency of body storage (%)	2.5	2
F) Reference body weight (kg)	Males: 76	Males: 68.1
	Females: 61	Females: 58.5
G) Constant	10^3^	10^3^
Calculated average requirement (rounded to nearest 5 (IOM) or 10 (EFSA))	Males: 625 RE/d	Males: 570 RE/d
	Females: 500 RE/d	Females: 490 RE/d
CV (%)	0.20	0.15
RDA (IOM) and PRI (EFSA) (rounded to nearest 100 and 50, respectively)	Males: 900 RE/d	Males: 750 RE/d
	Females: 700 RE/d	Females: 650 RE/d

EFSA, European Food Safety Administration.

### Knowledge gaps

With exception of the cited data in the EFSA recommendations, the data underlying estimation of vitamin A requirements are largely similar to the literature reviewed in NNR 2012. There is still substantial uncertainty in the variation of average requirements across populations, and the literature search did not indicate that new data have been published in this regard. One study from the US shows that intakes as low as 500 RE/d maintain adequate liver stores at 20 μg/g liver or 0.07 μmol/L in a study based on 34 women (39). However, it should be noted that dietary vitamin A intake excluding supplement users did not correlate with total body stores, and these challenges remain prevalent for the estimation of average requirements and recommended intakes of vitamin A. We note that dietary vitamin A intake may become excessive in some age groups among children in some Nordic and Baltic countries. Little data are available on excessive intakes among children and adolescents and health outcomes in the future, and no systematic reviews were available on this subject from the literature search. This could serve as a research priority for future evaluations of requirements and tolerable upper intake levels of vitamin A.

With respect to fracture risk and osteoporosis, there is a substantial lack of consensus regarding the role vitamin A may have on the skeleton in humans. The discrepancies between studies may be explained by several factors such as difficulties to analyze vitamin A status based upon serum analyses of retinol and retinyl esters and the inherent uncertainty in dietary intake assessment as well as measurements of different endpoints (i.e. bone mineral density vs fracture) and at different skeletal sites. Studies are also heterogeneous in terms of numbers of subjects, sex, and age. Another potentially important issue is that there is often a substantial lag time between exposure assessment and analyses of bone mineral density and fracture. There is clearly a need for studies in which the risk of hip fracture is studied in relation to vitamin A intake and serum levels in large cohorts with large numbers of fractures, making it possible to stratify results in relation to sex and age. Currently, it is difficult to know which levels of vitamin A are beneficial or harmful for the skeleton.
